# A novel classification method for NSCLC based on the background interaction network and the edge-perturbation matrix

**DOI:** 10.18632/aging.204004

**Published:** 2022-04-09

**Authors:** Yuan Tian, Caiqing Zhang, Wanru Ma, Alan Huang, Mei Tian, Junyan Zhao, Qi Dang, Yuping Sun

**Affiliations:** 1 Somatic Radiotherapy Department, Shandong Second Provincial General Hospital, Shandong Provincial ENT Hospital, Jinan, Shandong 250023, PR China; 2 Department of Respiratory and Critical Care Medicine, Shandong Second Provincial General Hospital, Shandong Provincial ENT Hospital, Shandong University, Jinan, Shandong 250023, PR China; 3 Department of Blood Transfusion, Beijing Hospital, National Center of Gerontology, Institute of Geriatric Medicine, Chinese Academy of Medical Sciences, Beijing, PR China; 4 Department of Oncology, Jinan Central Hospital, The Hospital Affiliated with Shandong First Medical University, Jinan, Shandong 250013, PR China; 5 Respiratory Department, Affiliated Hospital of Shandong University of Traditional Chinese Medicine, Jinan, Shandong 250014, PR China; 6 Nursing Department, The First Affiliated Hospital of Shandong First Medical University and Shandong Provincial Qianfoshan Hospital, Jinan, Shandong 250014, PR China; 7 Phase I Clinical Trial Center, Shandong Cancer Hospital and Institute, Shandong First Medical University and Shandong Academy of Medical Sciences, Jinan, Shandong 250012, PR China

**Keywords:** multi-dimensional, multi-omics, gene interaction, perturbation subtype, NSCLC

## Abstract

The biological functional network of tumor tissues is relatively stable for a period of time and under different conditions, so the impact of tumor heterogeneity is effectively avoided. Based on edge perturbation, functional gene interaction networks were used to reveal the pathological environment of patients with non-small cell carcinoma at the individual level, and to identify cancer subtypes with the same or similar status, and then a multi-dimensional and multi-omics comprehensive analysis was put into practice. Two edge perturbation subtypes were identified through the construction of the background interaction network and the edge-perturbation matrix (EPM). Further analyses revealed clear differences between those two clusters in terms of prognostic survival, stemness indices, immune cell infiltration, immune checkpoint molecular expression, copy number alterations, mutation load, homologous recombination defects (HRD), neoantigen load, and chromosomal instability. Additionally, a risk prediction model based on TCGA for lung adenocarcinoma (LUAD) and lung squamous cell carcinoma (LUSC) was successfully constructed and validated using the independent data set (GSE50081).

## INTRODUCTION

Smoking cessation and advancements in early detection and treatment [1], have resulted in a continuous decline in cancer death rates worldwide from 1991 to 2018, a total decline of 31%, particularly for lung cancer [2]. Lung cancer, on the other hand, remained the leading cause of cancer death in 2020, with an estimated 1.8 million deaths (18%) [3]. As is well known, NSCLC is the leading cause of cancer death in patients with lung cancer [4]. Thus, further investigation of disease biology, pathological type, genotype, application of predictive biomarkers, and treatment improvements for NSCLC are required.

Tumor heterogeneity is prevalent and plays a significant role in the progression of the disease [5, 6]. This heterogeneity can manifest itself in the uneven distribution of tumor cell subpopulations across the tumor (spatial heterogeneity) [7], or in the temporal variation in the molecular composition of cancer cells (temporal heterogeneity) [5, 8], both of which present difficulties for clinical research [9]. It has been suggested that expression profiles measured at different time points or under different conditions may exhibit significant temporal heterogeneity [5, 8, 9]. However, the biological functional network of tumor tissues remains relatively stable over time and under a variety of conditions, effectively avoiding the effects of tumor heterogeneity [10–14].

Based on edge perturbation [15–18], functional gene interaction networks were used to deduce the pathological environment of NSCLC patients at the individual level [19–22], and to identify cancer subtypes with the same or similar status, followed by a multi-dimensional and multi-omics comprehensive analysis for validation [23–26].

## MATERIALS AND METHODS

### Published data sets

Lung adenocarcinoma (LUAD) and Lung squamous cell carcinoma (LUSC) expression data of the TCGA database, including clinical characteristics and survival information, were downloaded from the UCSC Xena website (https://xenabrowser.net/datapages/). Copy number variation (CNV) information was downloaded by R package RTCGA, while mutation data was downloaded by R package TCGAbiolinks. 986 (497/489) cancer samples with expression data, clinical characteristics, and survival information were prepared for the subsequent analysis. The basic clinical information of cancer samples from the TCGA dataset was displayed in Table 1. The flow diagram was provided in (Supplementary Figure 1).

**Table 1:** Clinical characteristic of TCGA-NSCLC patients.

**Parameter**	**Subtype**	**TCGA (LUAD + LUSC)**
Age	≥60	240
	<60	746
Stage	I	506
	II	274
	III	163
	IV	32
	Unknown	11
Gender	Female	396
	Male	590
Smoke_year	1	89
	2	249
	3	210
	4	403
	5	9
	Unknown	26
EGFR_mutation	NO	458
	YES	100
	Unknown	428
M	M0	733
	M1	31
	MX	214
	Unknown	8
N	N0	634
	N1	220
	N2	109
	N3	7
	NX	15
	Unknown	1
T	T1	277
	T2	553
	T3	112
	T4	41
	TX	3

The expression data of 288 lung control samples from the GTEX database were gotten from the UCSC Xena website (https://xenabrowser.net/datapages/). 181 NSCLC samples with survival information of the GSE50081 data cohort were downloaded from the GEO database (https://www.ncbi.nlm.nih.gov/geo/) and used as the validation set for model verification.

### Data preprocessing

In order to keep the data consistency, 70% of the genes with 0 expression level in the three data cohorts (TCGA-LUAD, TCGA-LUSC, GTEX-LUNG) were filtered out first, and then the R package sva was used for batch calibration.

### Construction of the background interaction network and edge-perturbation matrix

The Cytoscape plug-in ReactomeFIPlugIn was used to download the gene affiliation data of pathway coding in Reactome, and the functional gene interaction network in the database was constructed based on the existing gene or protein interaction information. Specifically, the background interaction network is a gene interaction network based on the pathway in Reactome, including protein-protein interaction, gene co-expression, protein domain interaction, GO (Gene Ontology) annotation, and proteins interaction data obtained from the text data mining analyses. The ReactomeFIPlugIn plug-in was used to download all genetic interaction pairs in the pathway and then merged into a large background network. The construction process of the edge disturbance matrix mainly included the following three steps [15].

First, according to the inconsistency of the expression levels of genes in the background interaction network between cancer and normal samples, the ranks of gene expression in cancer and normal samples were obtained separately (ranked from small to large, the smaller value of the expression level ranked in the front of the queue, while the bigger value of the expression level ranked in the back of the queue), and then the gene expression matrix was converted into a gene expression rank matrix.

Second, according to the interaction relationship of gene pairs in the background of interaction network, if two genes interaction existed, there would be an edge in the network that connected the two genes, then the difference between the ranks of this edge in the two genes was calculated. In this way, the delta rank matrix of each edge among all the samples were obtained. The calculation formula was displayed in the followings:


$$\delta_{e,s}=r_{i,s}-r_{j,s}$$

In the formula, “r_*i,s*_” represents the rank of gene “i” in sample “s”, “δ_*e,s*_” represents the delta rank of edge “e” in sample “s”, and gene “i” and “j” are connected by the edge “e”.

Third, the average expression level of genes in normal samples was converted into a normal sample gene expression rank matrix, and the average delta rank matrix (Benchmark delta rank vector) of normal samples was established according to the background interaction network. Then, the delta rank matrix and the average delta rank matrix of the normal samples were used to construct the edge-perturbation matrix. The calculation formula was displayed in the followings:


$$\Delta_{e,s}=\delta_{e,s}-{\bar{\delta }}_{e}$$

In the formula, “δ_*e,s*_” represents the delta rank value of the edge “e” in the sample “s”, and ${\bar{\text{δ}}}_{e}$ represents the average delta rank of the delta rank matrix and the normal sample, which was the eigenvalue of the edge in the edge disturbance matrix.

Finally, the specific edge perturbation matrix in the cancer sample was screened. According to the Kruskal-Wallis test, the difference of the edge disturbance matrix between the normal and the cancer sample was calculated, and the top 30,000 different edges would be selected according to the level of significant difference; At the same time, the standard deviations (SDs) of the edge disturbance matrix in the cancer samples were calculated, and the top 30,000 different edges according to the SDs were selected. The intersection of the above two edges were considered as the specific edge perturbation matrix of the cancer samples, which was named as the characteristic edge. The feature logarithm conversion formula between cancer sample and feature sample was listed as follows:


$$features=\log_{2}(\Delta_{es}+1)$$

In the formula, “Δ_*es*_” is the eigenvalue of the edge in the edge disturbance matrix.

### Clustering and survival analysis of features of edge disturbance matrix

According to the feature edge of the edge perturbation matrix specific to the cancer sample, the R package ConsensusClusterPlus was used to perform the consistent clustering analysis. The distance used for clustering is spearman, the clustering method is pam, and 100 repetitions are performed to ensure the stability of the classification.

Log-rank test was used to explore the difference in survival time among subtypes, and R package survival was used to draw the KM survival curve of patients subtypes. The R package clusterRepro and independent data sets were used to verify the efficacy of clustering of the feature edge perturbation matrices. Then, the intra-group proportion (IGP) of each subtype would be calculated, which the larger IGP indicated the better the consistency in the clustering group.

### Feature analysis of edge disturbance feature subtypes

Based on the known literature or calculated various feature indicators, statistical tests were used to explore the correlation between edge disturbance feature subtypes and known feature indicators. Tumor purity and ploidy were derived from TCGA data pan-cancer analysis [27].

The homologous recombination defect score (HRD score), neoantigen load, and genome alteration frequency were derived from previous studies on the analysis of immune characteristics of TCGA data [28]. Based on previous studies, the patient's stemness index (mRNAsi) and epigenetically regulated-mRNAsi (EREG-mRNAsi) were obtained [11].

The three indicators of chromosomal instability (LST score, TAI score, and LOH score) were derived from previous studies based on the correlation analyses between genome damage and homologous recombination defects [29]. The R package CIBERSORT was used to calculate the infiltration scores of 22 immune cell types. The expression level of immune checkpoint molecules is the RNAseq level of cancer samples currently used.

In the significance analysis between various values (expression, infiltration ratio, mutation count, etc.), the Wilcoxon test was used to compare the differences between the two sets of samples. In the graphical display, ns (no significance) represents *p* > 0.05, ^*^represents *p* ≤ 0.05, ^**^represents *p* ≤ 0.01, ^***^represents *p* ≤ 0.001, and ^****^represents *p* ≤ 0.0001.

### Copy number variant (CNV) analyses

The GISTIC method was used to detect the common copy number alteration area in all samples based on the SNP6 CopyNumber segment data. The parameters of the GISTIC method were set as follows: Q ≤ 0.05 was taken as the significance standard of the alteration. 0.90 was adopted as the confidence level, when the peak interval was determined. The analysis was performed through the corresponding MutSigCV module of the online analysis tool GenePattern (https://cloud.genepattern.org/gp/pages/index.jsf) developed by Broad Research Institute.

### TIDE (tumor immune dysfunction and exclusion) prediction

The TIDE (http://tide.dfci.harvard.edu/) analysis tool was developed by researchers from Harvard University and used to evaluate the clinical effects of immune checkpoint suppression therapy. A higher tumor TIDE prediction score was corresponded to a lower immune checkpoint suppression therapy efficacy and poor prognosis. The prediction for the prognosis of immune checkpoint inhibitor (ICI) treatment in this analysis was completed by the TIDE score.

### Subtype-specific characteristic clusters and pathway enrichment of feature clusters

The unique biological functions and pathways of the subtype were analyzed by identifying the unique characteristic clusters of the subtype, and Z-score was used to standardize the characteristic values of the characteristic edges, and then the characteristic clusters were screened through the following steps.

The first step is to perform hierarchical clustering based on the normalized characteristic values of the characteristic edges, and the clustering method was “complete linkage”. The number of clusters is 100, and clusters with fewer than 30 characteristic edges would be filtered out. Second, for each remaining cluster, the percentage of “characteristic edges”, whose “absolute value” of the perturbation mean was greater than 0.5 to all the “characteristic edges”, would be calculated. Third, the cluster with an average percentage greater than 0.7 in a subtype would be considered as the characteristic cluster of that subtype, and all genes in the characteristic edge corresponding to the characteristic cluster would be used for pathway enrichment analysis. Online software Metascape (http://metascape.org) would be used for enrichment analysis of KEGG and Reactome pathways, setting the parameter *P* < 0.01.

### Differential expression analysis and differential methylation site recognition

The R software package limma would be used for analyzing the expression profile and methylation level of the subtype samples. According to the fold change (|logFC|) and significant False Discovery Rate (FDR), the genes and methylation sites that were differentially expressed in the subtype samples were screened.

### Prognostic analysis

Univariate Cox regression analysis would be used for determining the hazard ratio (HR) and prognostic significance of different expressed genes, and genes with *p* < 0.01 would be screened as prognostic-related genes. The Kaplan-Meier method was used to generate the survival curve for prognostic analysis, and the log-rank test was used to determine the significance of the difference. The receiver operating characteristic curve (ROC) was used to evaluate the risk model’s prediction of the score, and the area under the curve (AUC) was quantified by the R package survivalROC.

### Ethics statement

No interaction with human subjects of the study was involved, no ethical issues were encountered, and no ethical approval was needed.

## RESULTS

### Background interaction network construction and gene expression extraction

The Cytoscape plug-in ReactomeFIPlugIn was used to download the gene affiliation data of pathway encoding in Reactome. According to the existing gene or protein interaction information, including protein-protein interaction, gene co-expression, protein domain interaction, GO annotation and protein interaction data based on text mining, integrating all interaction information, the functional background interaction network would be constructed. A total of 7,360 nodes and 169,710 edges were included in the background interaction network.

In the three data sets (TCGA-LUAD, TCGA-LUSC, and GTEX-LUNG), 70% of the genes whose expression levels were 0 were filtered out, and then the R package sva was used for batch correction. The two datasets TCGA-LUAD and TCGA-LUSC were merged as the expression profile of cancer samples (26209 genes ×986 samples), and the GTEX-LUNG dataset was used as the expression profile of normal samples (26209 genes ×288 samples).

After filtering out the nodes in the background interaction network that were not within the range of 26209 genes, a new background interaction network was constructed, which included 6327 nodes and 153314 edges, which would be used for subsequent calculation of edge disturbances Feature matrix. The interaction network was closely connected, and most of the nodes in the network had a high degree. According to the degree of nodes in the background interaction network, the nodes were sorted from large to small.

### Perturbation matrix (edge-perturbation matrix) construction and feature extraction

The perturbation of the edge in the background interaction network would inevitably lead to the alteration of the interaction relationship in the network, and the perturbation of the gene in the network to the edge could be reasonably used for simulating and revealing the pathological environment at the individual level. In order to measure the degree of disturbance of the background interaction network at the level of a single sample, based on the difference in expression fluctuations between cancer and normal samples, the Edge-Perturbation Matrix (EPM) was constructed separately. In order to compare the difference in EPM between cancer and normal samples, 1000 features were randomly selected, and logarithmic transformation was performed. It was found that the feature values of cancer samples were significantly higher than those of normal samples (Supplementary Figure 2). This showed that the degree of edge perturbation in cancer samples was greater, indicating that the degree of perturbation in the background network of cancer samples was much more obvious than that of normal samples. This provided reliable evidence for the subsequent use of EPM to reveal the heterogeneity among NSCLC samples.

In order to further perform feature extraction in this study, we used the Kruskal-Wallis test to calculate the difference of edge perturbations between cancer and normal samples, and calculated the Standard Deviations (SDs) of the edge perturbation matrices in cancer samples. The top 30,000 different edges of the above two methods were selected respectively, and the intersection edges (*N* = 5468) were selected as the feature edges of the edge perturbation matrix in the cancer sample for subsequent analyses.

### Clustering and survival analyses of edge disturbance matrix features

**Figure 1:**
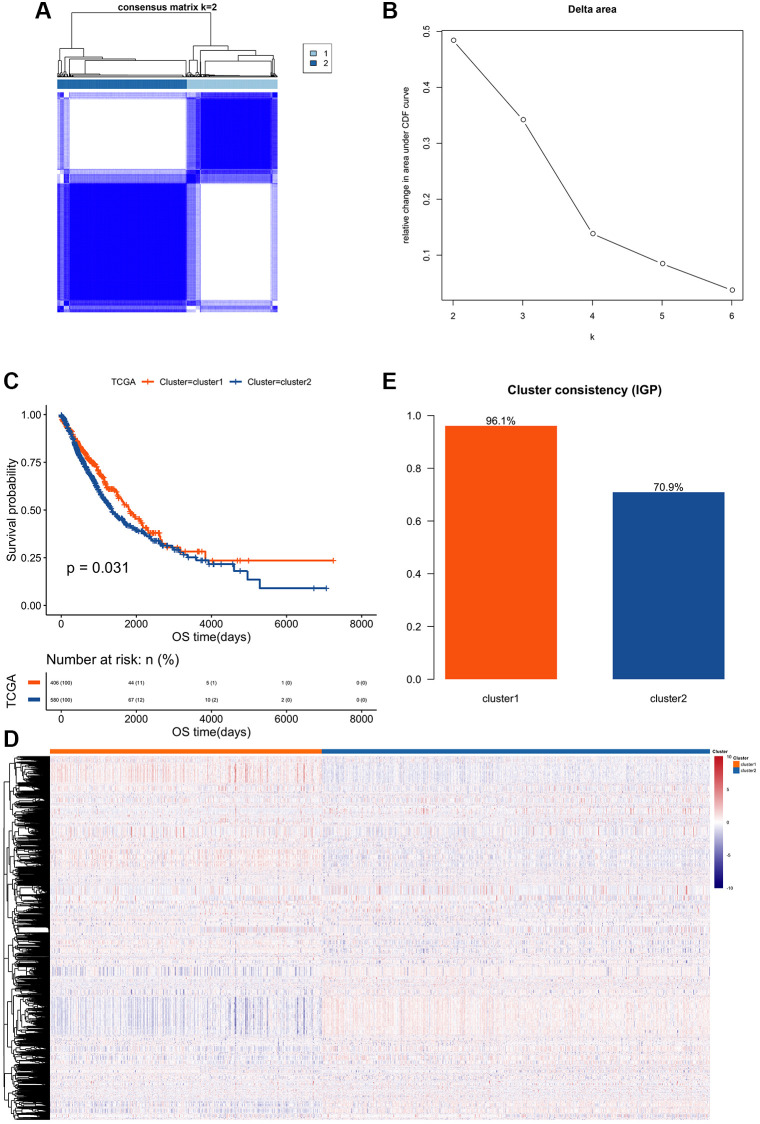
**Clustering and survival analysis of feature edge perturbation matrix.** (**A**) Based on the extracted 5468 feature edges, a consistent cluster analysis was performed according to their feature values, and 986 cancer samples were divided into two different subtypes. (**B**) Cumulative Distribution Function of Consistent Cluster Analysis. The abscissa axis represents the K value; the ordinate axis represents the relative change in area under CDF curve. (**C**) The prognostic survival curves of the two cluster subtypes. The abscissa axis represents the overall survival time; the ordinate axis represents the survival probability corresponding to different survival time points. (**D**) Z-score heatmap of eigenvalues of feature edges: Using hierarchical clustering method to perform cluster analyses on the extracted feature values, it is found that there are obvious specific feature differences between the two subtypes. (**E**) Validation of clustering performance of gene interaction perturbation: Using an independent data set (GSE50081) to verify the clustering performance of edge perturbation features. The larger IGP corresponds to the better consistency of the clustering group.

Based on the 5468 feature edges extracted in the previous step, consistent clustering analyses were put into practice based on their feature values. 986 cancer samples were divided into two different subtypes (Figure 1A and 1B). These two subtypes were named cluster 1 (*N* = 406) and cluster 2 (*N* = 580), which displayed significant differences in prognosis from each other (Figure 1C). The distance used for clustering was spearman, the clustering method was pam, and 100 repetitions were performed. The hierarchical clustering method was used to perform cluster analysis on the extracted features, and it was found that there were obvious specific feature in subtypes (Figure 1D).

In order to verify the clustering performance of gene interaction perturbation, we collected a set of independent expression data sets (GSE50081) from reported studies, and used the R package clusterRepro to calculate the intra-group proportion (IGP) of each subtype. The results showed that both cluster 1 and cluster 2 had higher IGP values (Figure 1E), which indicated that the clustering consistency of the cluster analysis based on the edge perturbation matrix in this study was better.

### Comparative analysis of edge perturbation feature subtypes

Genomic heterogeneity indicators were obtained from reported studies, and statistical tests were used to explore their differences in edge perturbation feature subtypes. The results showed that the cluster 2 subtype with poor prognosis displayed higher tumor purity and genome ploidy compared with cluster 1 (Supplementary Figure 3A and 3B).

Based on previous analyses of TCGA samples, the transcriptome (mRNAsi) and epigenetic regulatory (EREG-mRNAsi) index of NSCLC samples were obtained. Through the evaluation of the stemness index in subtypes, it was found that the cluster 2 displayed a higher stemness index (Supplementary Figure 3C and 3D). The clinical characteristics of the two subtype samples were statistically analyzed. Fisher’s exact test results were displayed in the form of Stage, *T*, *N* staging and Age (*p* < 0.05, Supplementary Figure 3E–3J).

**Figure 2:**
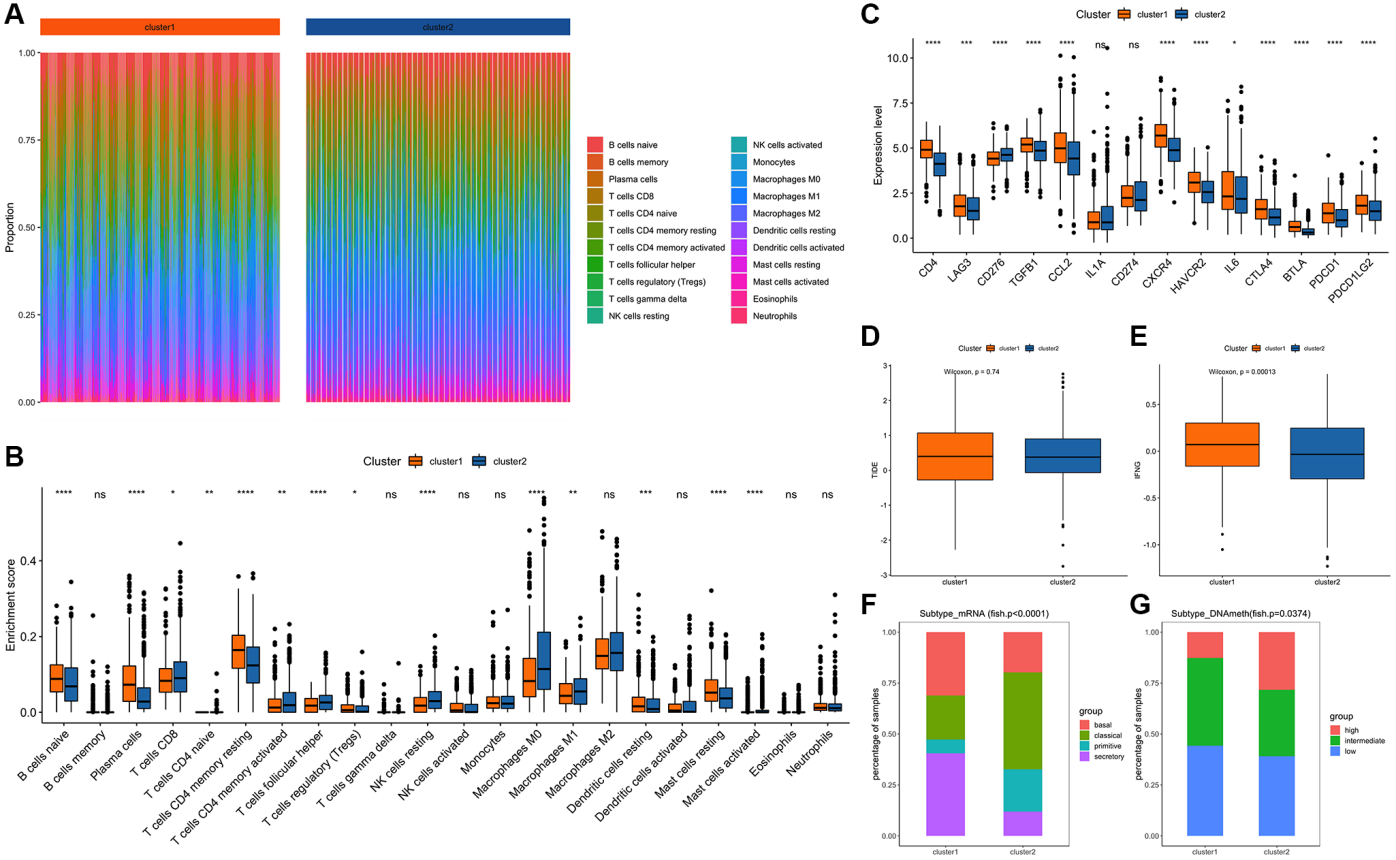
**Comparison and analysis of feature subtypes of edge perturbation.** (**A**) Infiltration scores of 22 immune cells in different samples: Different colors represent different immune infiltrating cells. The abscissa axis represents different samples; the ordinate axis represents the proportion of different immune cells. (**B**) Comparison of the differences of 22 immune cell infiltration scores among different cluster subtypes: The abscissa axis represents different types of immune cells; the ordinate represents the proportion of infiltrated immune cells. (**C**) Comparison of the expression levels of important immune checkpoint molecules among different cluster subtypes. (**D**) TIDE comparison between cluster 1 and cluster 2: The abscissa axis represents different clusters; the ordinate axis represents TIDE. (**E**) IFNG comparison between cluster 1 and cluster 2: The abscissa axis represents different clusters; the ordinate axis represents IFNG. (**F**) Comparison of mRNA expression levels among different subtypes: The different colors represent the known pathological types of NSCLC. The abscissa axis represents different clusters, and the ordinate axis represents the expression level of mRNA. (**G**) Comparison of DNA methylation levels among different subtypes: The different colors represent the levels of DNA methylation. The abscissa axis represents different clusters, and the ordinate axis represents the proportion of samples with different levels of DNA methylation.

The infiltration scores of 22 immune cell types were calculated by the R package CIBERSORT, and the differences among subtype samples were further explored. The results showed that immune cells such as B cells naive, Plasma cells and Mast cells resting were significantly different in the two subtypes (*p* < 0.05, Figure 2A and 2B). On the other hand, the expression differences of important immune checkpoint molecules of the two subtypes were also analyzed, and it was found that multiple immune checkpoint molecules, such as PDCD1, CD4, and LAG3, were significantly different between the two subtypes (Figure 2C). Furthermore, based on online verification (http://gepia.cancer-pku.cn/detail.php?gene), CD276, CXCR4, and BTLA were found to be significantly related to the prognosis of LUAD, while CCL2 was significantly related to the prognosis of LUSC.

TIDE was used to evaluate the clinical efficiency of two subtypes receiving immune checkpoint inhibitors. There was no significant difference in TIDE scores between the two subtypes, but there were significant differences for the expression of IFNG immune checkpoints (Figure 2D and 2E).

**Figure 3:**
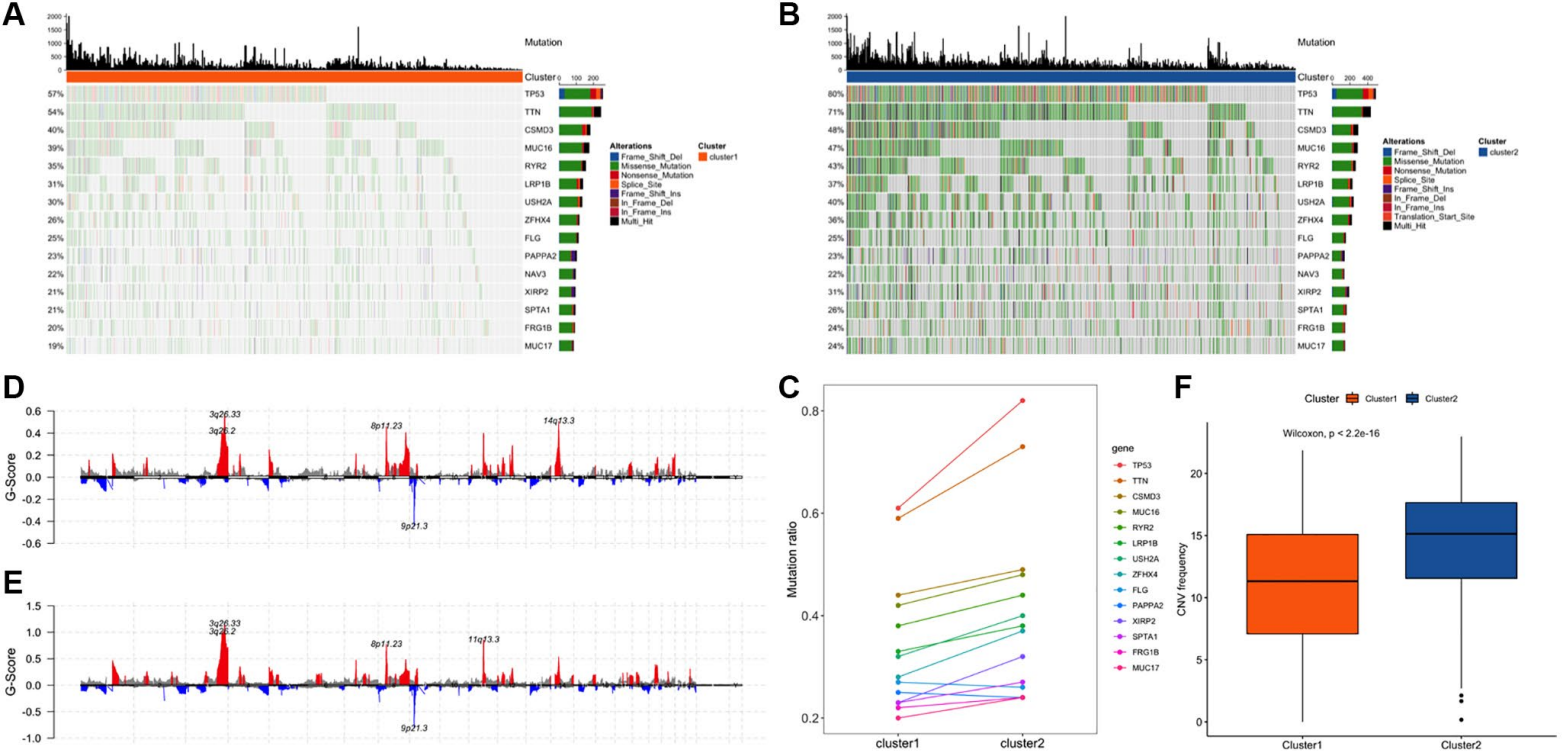
**Comparison of genome alterations between cluster 1 and cluster 2.** (**A**) The distribution of 15 mutated genes in cluster 1 among the co-occurring genes with mutation frequencies in the top 20 of the two clusters. (**B**) The distribution of 15 mutated genes in cluster 2 among the co-occurring genes with mutation frequencies in the top 20 of the two clusters. (**C**) Comparison of the mutation ratios of 14 genes in the two clusters. (**D**) Distribution of concentrated copy number amplification and deletion regions in the cluster 1. (**E**) Distribution of concentrated copy number amplification and deletion regions in the cluster 2. (**F**) Frequency distribution of copy number variation among subtype samples.

Comparing the edge perturbation feature subtypes with the reported subtypes of NSCLC, the fish results showed that the distribution of cancer subtypes corresponding to the edge perturbation feature subtypes were different (Figure 2F and 2G). To further explore the molecular differences between the two sample sets, the R package maftools was used for somatic mutations analysis. Among the top 20 genes with mutation frequencies in the two groups, the distribution of the 15 common mutation genes that appeared in both of them were shown in (Figure 3A and 3B). In order to observe the proportion of high-frequency mutation genes in the subtypes in detail, a line chart of the proportions of these 15 genes between the two subtypes was drawn and displayed in (Figure 3C). The distribution of CNV in the two sets of samples was shown in (Figure 3D and 3E), which indicated that the CNV frequency of the two sets was significantly different (*p* < 0.05, Figure 3F).

In order to further investigate the correlation between the innate immune escape mechanism and subtypes, we compared some potential factors that determine tumor immunogenicity of the two subtypes, including tumour mutational load (TML), homologous recombination deficiency (HRD), and neoantigen load, chromosome instability (Supplementary Figure 4). The functional enrichment results of the two subtype-specific clusters were further analyzed. The cluster 1 was found to be significantly enriched in those pathways such as Peptide chain elongation and Translation initiation complex formation, while the cluster 2 was significantly enriched in Fc epsilon receptor (FCERI) signaling, Fcgamma receptor (FCGR) dependent phagocytosis and other pathways (Supplementary Figure 5A and 5B).

### Identification of differential methylation sites among characteristic subtypes

The TCGA-LUAD and TCGA-LUSC methylation value matrices were merged, and the samples (*N* = 797) that appeared in the two subtypes would be retained, and then more than 70% of the samples with NA sites were filtered out and filled with 0. Finally, after filtering out and filling, 395786 methylation sites were collected for differential analyses.

According to the fold change (|logFC|>0.1) and the significance threshold (FDR<0.01), a total of 7304 differentially methylated sites were screened in the two subtype samples by the R package limma. The |logFC| values of the differentially methylated sites were sorted from big to small, and the top 200 differentially methylated sites were plotted (Supplementary Figure 5C).

### Construction and verification of risk scoring models based on differentially expressed prognostic-related genes

**Figure 4:**
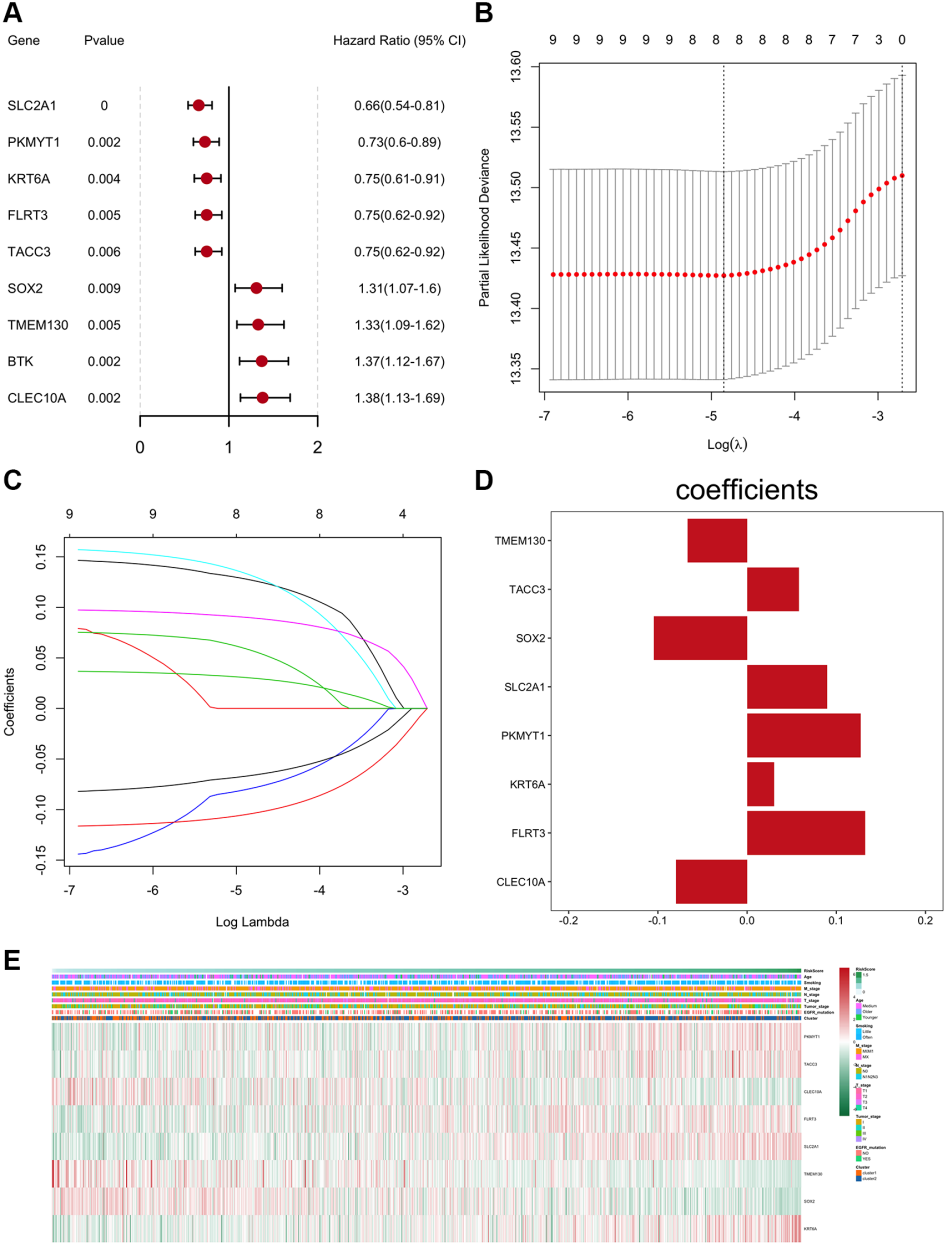
**Construction and verification of the prognostic-related gene risk scoring model.** (**A**) The forest plot of prognostic genes (univariate cox analysis result). (**B**) Confidence interval of each Lambda corresponding to LASSO regression. (**C**) The change trajectory of the independent variable in LASSO regression; the abscissa represents the logarithm of the independent variable Lambda, and the ordinate represents the coefficient of the independent variable. (**D**) LASSO regression coefficient of key prognostic genes. (**E**) Z-score heatmap of key prognostic genes expression levels.

According to the fold change (|logFC|>0.585) and the significance threshold (FDR<0.01), a total of 945 differentially expressed genes were screened from the two subtype samples by the R package limma (Supplementary Figure 5D and 5E). Univariate cox regression analysis was performed on these 945 differentially expressed genes. When *P* value was less than 0.01, 9 differentially expressed genes were found to be related to the prognosis, and a forest plot was shown in (Figure 4A).

The LASSO method was further used to screen out 8 key prognostic genes (Figure 4B–4E), and after weighting the expression of these 8 genes by the LASSO regression coefficient, a risk scoring model for predicting the survival of the sample was constructed (“exp” represents gene expression level, “coef” represents LASSO regression coefficient).


RiskScore=∑exp × coef

**Figure 5:**
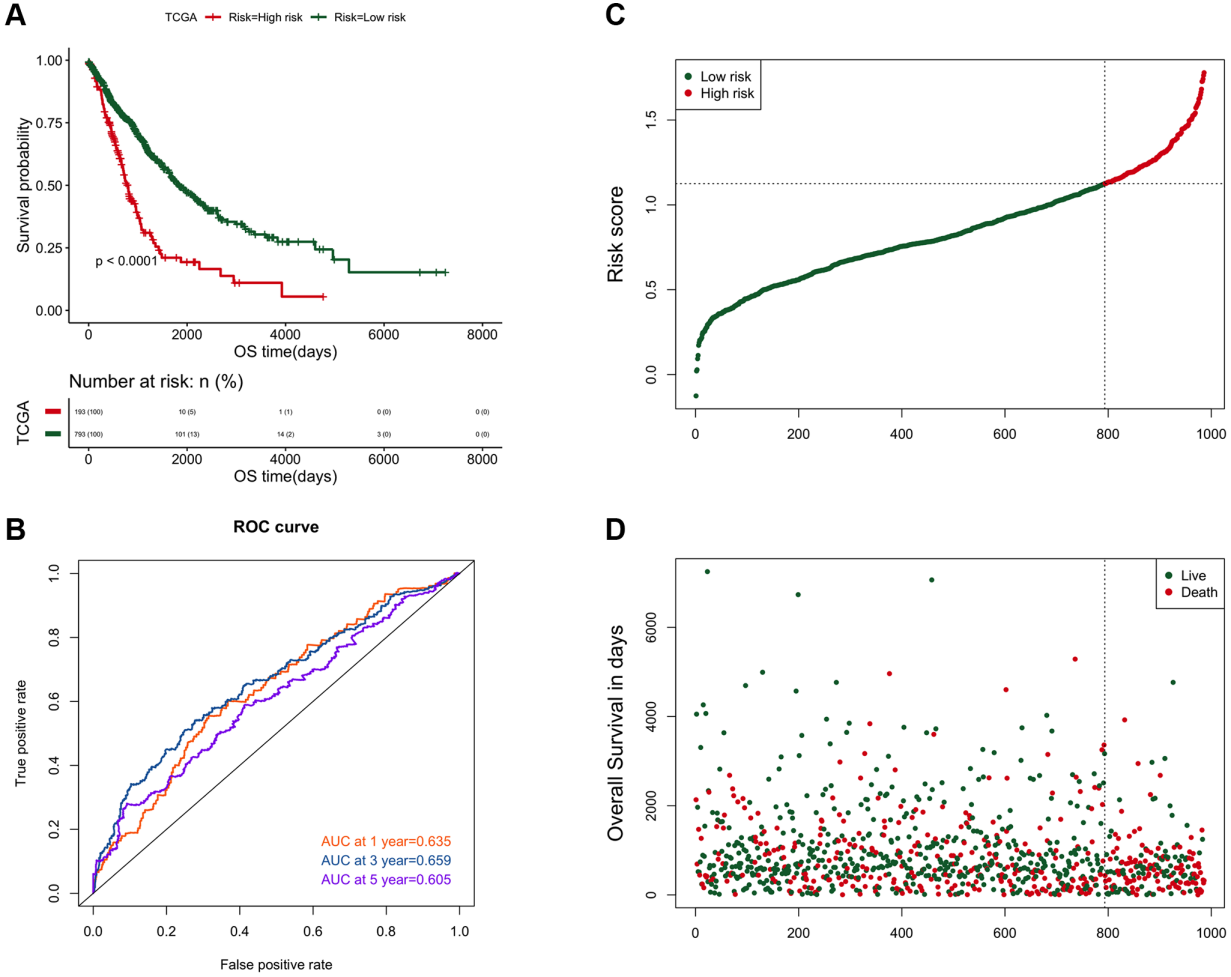
**The efficiency analysis results of the risk prediction model.** (**A**) Comparison of the prognosis between high and low risk groups. The abscissa axis represents the survival time (days); the ordinate axis represents the survival probability. (**B**) AUC curve of risk score on 1, 3, and 5-year survival prediction efficiency. (**C**) Risk score curve of the two groups. The abscissa axis represents the number of samples; the ordinate axis represents the risk score. (**D**) The ranking results of risk scores from small to large. The abscissa represents the number of samples, and the ordinate represents the survival time.

The risk score of each cancer sample was calculated based on the risk model. The surv_cutpoint function in the R package survminer was used to determine the classification threshold (1.1254), and further divide the cancer samples into high and low risk groups (*N* = 193/793), while significant prognosis differences were found in the two groups (Figure 5A). ROC was used to evaluate the predictive efficiency of the model, the AUC of cancer samples at 1, 3, and 5 years were 0.635, 0.659, and 0.605 (Figure 5B–5D).

**Figure 6:**
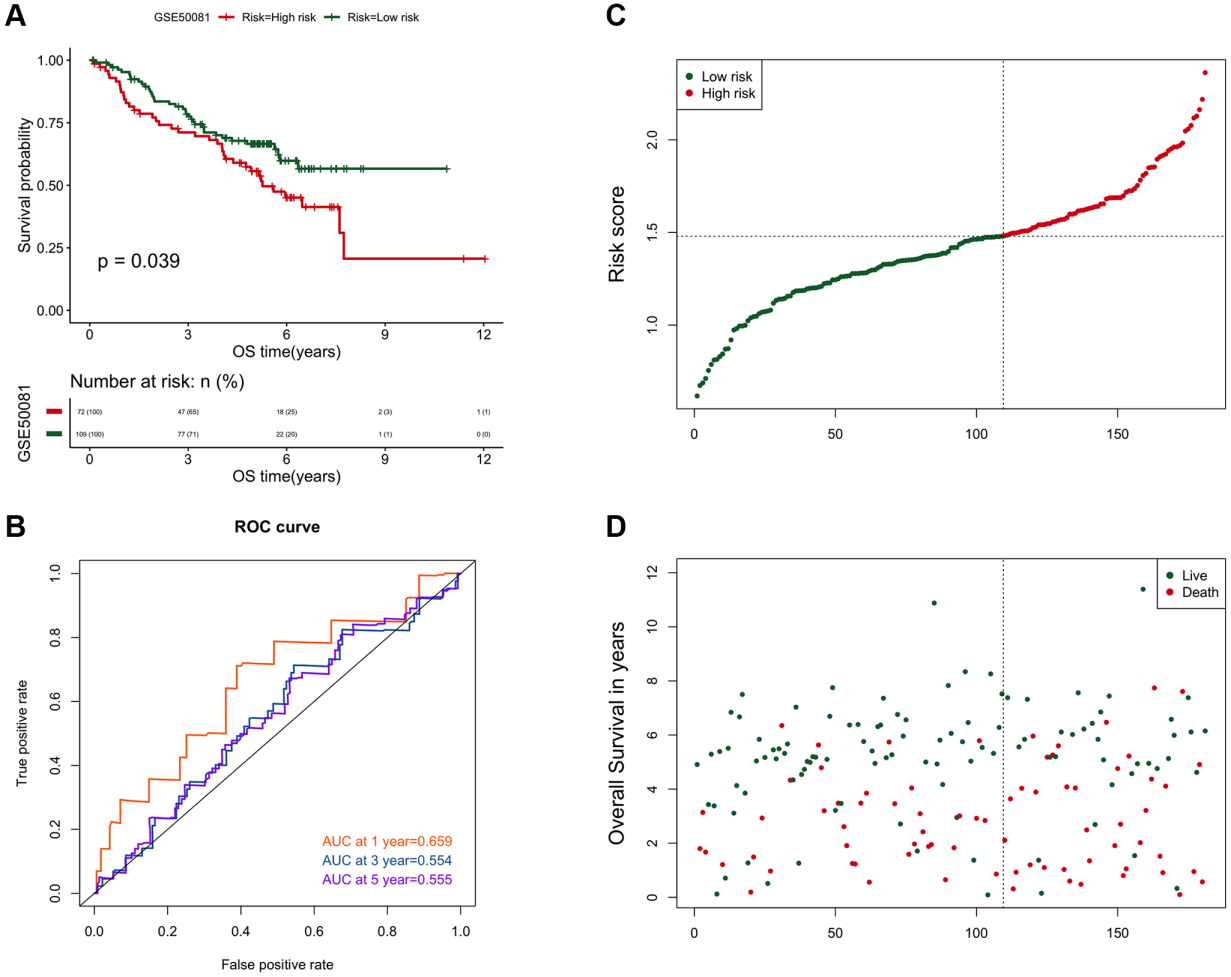
**The verification result of the risk scoring model in the independent data set GSE50081.** (**A**) Comparison of the prognosis between high and low risk groups in GSE50081. The abscissa axis represents the survival time (days); the ordinate axis represents the survival probability. (**B**) AUC curve of risk score on 1, 3, and 5-year survival prediction efficiency in GSE50081. (**C**) Risk score curve of the two groups in GSE50081. The abscissa axis represents the number of samples; the ordinate axis represents the risk score. (**D**) The ranking results of risk scores from small to large in GSE50081. The abscissa represents the number of samples, and the ordinate represents the survival time.

It was verified in the independent data set GSE50081, and a similar analysis result trend was obtained (Figure 6A). The ROC predictive efficiency AUC values at 1, 3 and 5 years were 0.659, 0.554 and 0.555 (Figure 6B–6D).

## DISCUSSION

Our understanding of cancer has promoted further with the advancement of sequencing technology and advances in cancer genome research [27, 28], particularly for lung cancers [27, 29–31]. These advances in genome research also enabled us to gain a comprehensive understanding of the temporal and spatial heterogeneity of lung cancer cells [29, 32], indicating that cancer's heterogeneity was unavoidable. However, recent research on biological functional networks has discovered that heterogeneity can be avoided or minimized to a certain extent through the use of sequencing and bioinformatics analysis technology [10–14]. Thus, using the R language-based bioinformatics analysis technology, we designed the following research to re-evaluate the alterations in the NSCLC genome after excluding tumor heterogeneity: Based on edge perturbation [15–18], functional gene interaction networks were used to deduce the pathological environment of individual patients with NSCLC [19–22], and to identify cancer subtypes with the same or similar status, followed by a multi-dimensional and multi-omics comprehensive analysis for validation [23–26].

After successfully constructing a background interaction network and extracting gene expression (Figure 1A and 1B), it was found that the characteristic value of the cancer sample was significantly higher than that of the normal sample (Figure 2A and 2B). This indicated that the degree of edge perturbation in cancer samples was greater, implying that the degree of perturbation in the background network of cancer samples was significantly greater than in normal samples [15], providing reliable evidence for the subsequent use of EPM to reveal the heterogeneity of NSCLC samples.

Cluster analysis was performed using the R package ConsensusClusterPlus, as described previously [33, 34], and two distinct cluster subtypes were identified: cluster 1 (*N* = 406) and cluster 2 (*N* = 580) (Figure 3A–3D). Additional validation using the data set GSE50081 revealed that the clustering consistency based on the characteristic clustering analysis of the edge perturbation matrix was improved (Figure 3E). In comparison to cluster 1, cluster 2 had a higher tumor purity, ploidy, and stemness index (Supplementary Figure 3A–3D). Additionally, significant differences between clusters 1 and 2 were observed in stage, *T*, *N*, age, 22 immune cell infiltration scores, and differential expression of immune checkpoint molecules and IFNG (Supplementary Figure 3E–3J, Figure 2A–2E), which largely correlated with patient prognosis (Supplementary Figure 3C) [35, 36]. When the edge perturbation characteristic subtypes were compared to the reported NSCLC subtypes, the distribution of cancer subtypes corresponding to the edge perturbation characteristic subtypes remained clearly distinct (Figure 2F and 2G). Further examination of the molecular differences between the two clusters revealed obvious differences in either the mutation frequency (Figure 3A–3C), or the copy number variation frequency and distribution for the 15 genes co-occurring in the top 20 (Figure 3D–3F). This confirmed the molecular distinctions between these two cluster subtypes. Based on the above findings, we hypothesized that EPM-based clustering subtype classification could be a novel classification method for adenosquamous carcinoma that was not inferior to the traditional pathological classification [37, 38].

TML, HRD, neoantigen load, and chromosome instability all played significant roles in the development of cancer [39–44]. As a result, these potential tumor immunogenicity-related factors were compared between the two cluster subtypes, and it was discovered that these indicators demonstrated statistically significant differences between the two clusters (Supplementary Figure 4). Similar differences were observed in analyses of the two subtype-specific clusters’ KEGG pathway enrichment and methylation site recognition (Supplementary Figure 5A–5C). As a result of the above findings, we hypothesized that the innate immune escape mechanism between the two subtypes may be significantly different, confirming the importance of classifying NSCLC using this method.

As previously reported [45–47], LASSO was used to construct a risk scoring model containing eight genes based on the differential expression of prognostic-related genes between the two clusters (Figure 4) [48]. Regardless of whether it was verified in those two clusters (Figure 5) or in external data (GSE50081) (Figure 6), the risk scoring model demonstrated high prediction efficiency. Due to the fact that the data and validation data for this prediction model were derived from clinical sequencing results, it was believed that the prediction model would have a high probability of clinical applicability. In other words, this further demonstrated the feasibility and rationality of the clustering analysis method based on EPM for the classification of NSCLC.

The EPM was constructed using the differences in expression fluctuations between cancer and normal samples, and then features for cluster analyses were extracted to accomplish the purpose of NSCLC classification. The advantage of this method is that it attempts to minimize the influence of tumor sample heterogeneity as much as possible, demonstrating some degree of innovation. With the rapid development of next-generation sequencing, for elderly patients with lung cancer, their blood samples can be used for next-generation sequencing, and then this method can be used for pathological typing, which can effectively avoid the damage and risks caused by pathological biopsy. Regardless of the method used to classify NSCLC or the risk scoring model developed, additional clinical samples and related basic experiments are still need to be conducted.

## CONCLUSION

Clustering analysis using EPM for NSCLC classification is a feasible typing method that minimizes the effect of cancer sample heterogeneity. The risk scoring model constructed using those two clusters and involving eight genes has a high prediction efficiency.

## Supplementary Materials

Supplementary Figures
