# Supplementary material for: A novel classification method for NSCLC based on the background interaction network and the edge-perturbation matrix
Source: Aging (Albany NY). 2022 Apr 9;14(7):3155–74. doi: 10.18632/aging.204004 (PMC9037255; doi:10.18632/aging.204004)
Supplement: Supplementary Figures [file aging-14-204004-s001.pdf]

SUPPLEMENTARY FIGURES

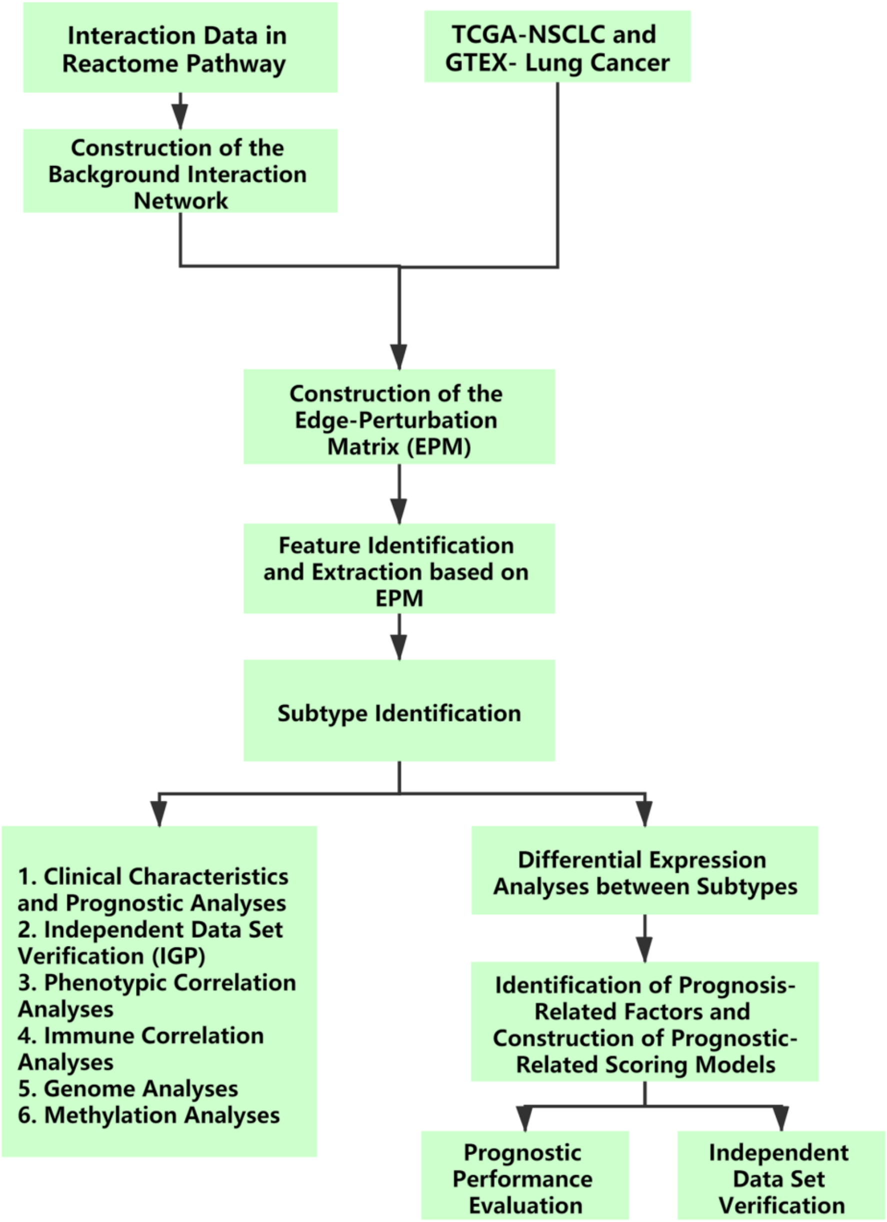

Supplementary Figure 1. The flow diagram of the study.

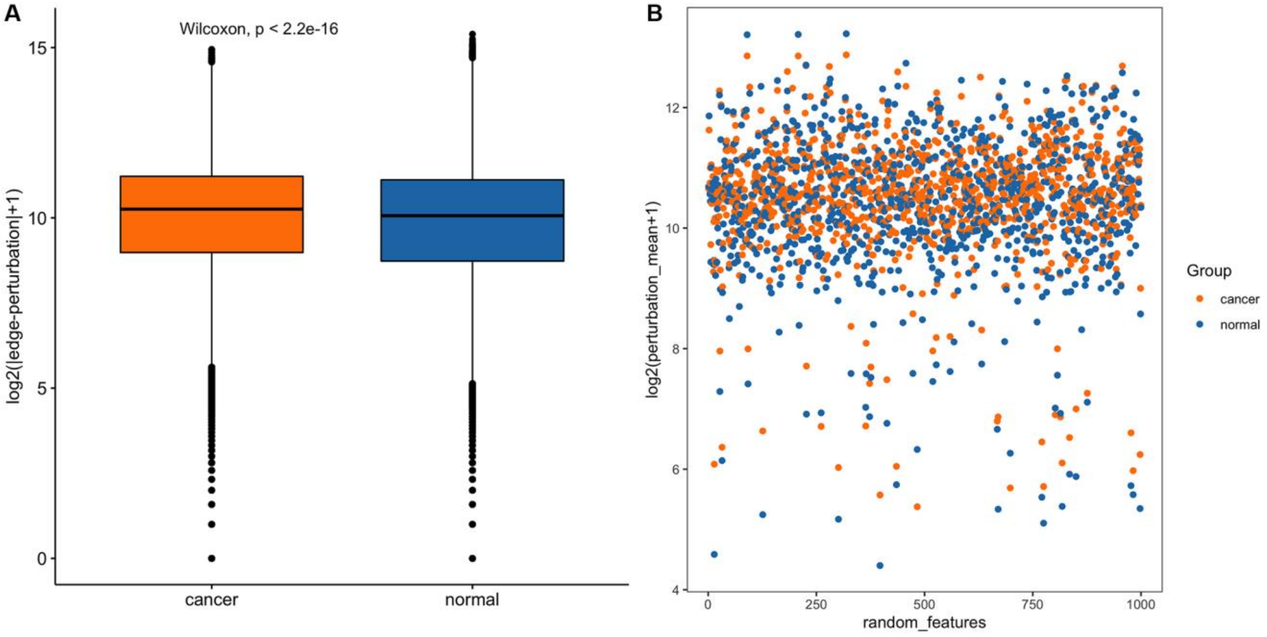

Supplementary Figure 2. Edge-perturbation matrix construction and feature extraction. (**A**) Box-plot diagrams of randomly selected 1000 features in cancer and normal samples. (**B**) Scatter plots of randomly selected 1000 features in cancer and normal samples.

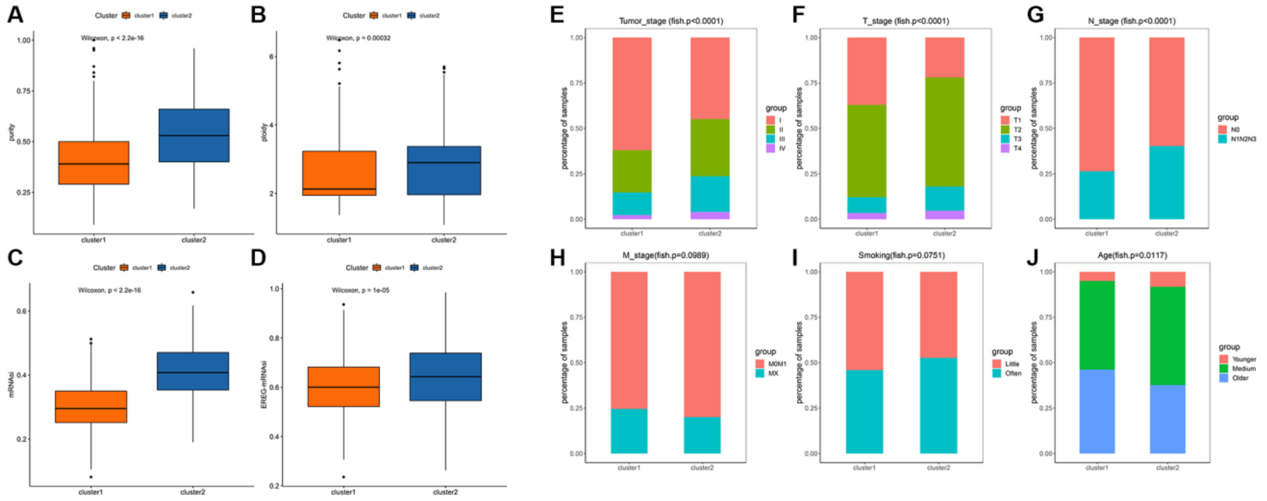

Supplementary Figure 3. (**A**–**D**) Comparative analysis results of edge perturbation feature subtypes. (**A**) Differences in tumor purity between cluster 1 and cluster 2: The abscissa axis represents the cluster group; the ordinate axis represents the percentage of tumor purity. (**B**) Differences in genome ploidy between cluster 1 and cluster 2: The abscissa axis represents the cluster group; the ordinate axis represents the percentage of tumor genome ploidy. (**C**) Differences in stemness indices of mRNA between cluster 1 and cluster 2: The abscissa axis represents the cluster group; the ordinate axis represents the percentage of stemness indices of mRNA. (**D**) Differences in epigenetic regulated stemness indices of mRNA between cluster 1 and cluster 2: The abscissa axis represents the cluster group; the ordinate axis represents the percentage of epigenetic regulated stemness indices of mRNA. (**E**–**J**) Comparison of clinical characteristics among different subtypes. (**E**) Comparison of the proportions of different stages (Stage I–IV) in different clusters: The abscissa axis represents different clusters; the ordinate axis represents the proportion of different stages. (**F**) Comparison of the proportions of different *T* stages (T1–T4) in different clusters: The abscissa axis represents different clusters; the ordinate axis represents the proportion of different T stages (T1–T4). (**G**) Comparison of the proportions of different N stages (N1–N3) in different clusters: The abscissa axis represents different clusters; the ordinate axis represents the proportion of different *N* stages (N1–N3). (**H**) Comparison of the proportions of different M stages (M0M1 or Mx) in different clusters: The abscissa axis represents different clusters; the ordinate axis represents the proportion of different M stages (M0M1 or Mx). (**I**) Comparison of the proportions of different smoking status in different clusters: The abscissa axis represents different clusters; the ordinate axis represents the proportion of different smoking status (little or often). (**J**) Comparison of the proportions of different age stages in different clusters: The abscissa axis represents different clusters; the ordinate axis represents the proportion of different age stages (younger, medium or older).

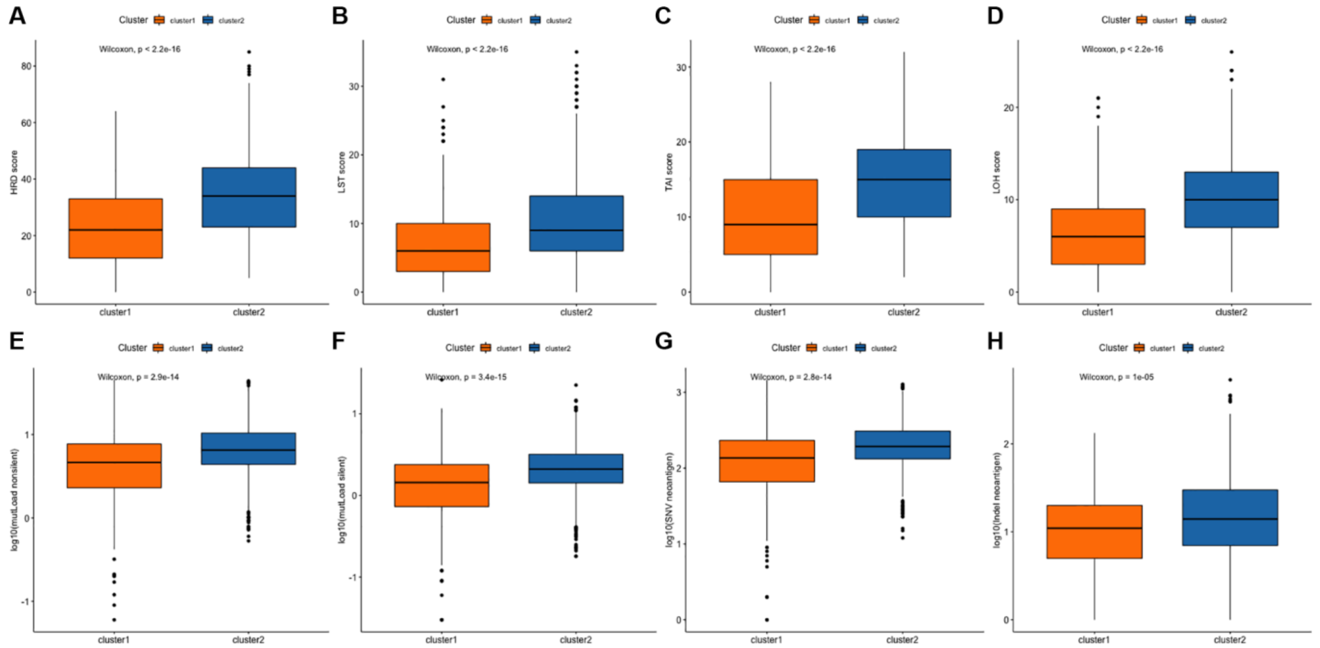

Supplementary Figure 4. Comparison of immune escape mechanism between the two clusters. (**A**) Comparison of homologous recombination deficiency scores between the two clusters: The abscissa axis represents different clusters; the ordinate axis represents the HRD score. (**B**) The level of chromosome instability between the two clusters: The abscissa axis represents different clusters; the ordinate axis represents the LST score. (**C**) The level of chromosome instability between the two clusters: The abscissa axis represents different clusters; the ordinate axis represents the TAI score. (**D**) The level of chromosome instability between the two clusters: The abscissa axis represents different clusters; the ordinate axis represents the LOH score. (**E**) The level of tumor mutation load between the two clusters: The abscissa axis represents different clusters; the ordinate axis represents the value of log10 (mutLoad nonsilent). (**F**) The level of tumor mutation load between the two clusters: The abscissa axis represents different clusters; the ordinate axis represents the value of log10 (mutLoad silent). (**G**) The level of tumor neoantigen load between the two clusters: The abscissa axis represents different clusters; the ordinate axis represents the value of log10 (SNV neoantigen). (**H**) The level of tumor neoantigen load between the two clusters: The abscissa axis represents different clusters; the ordinate axis represents the value of log10 (Indel neoantigen).

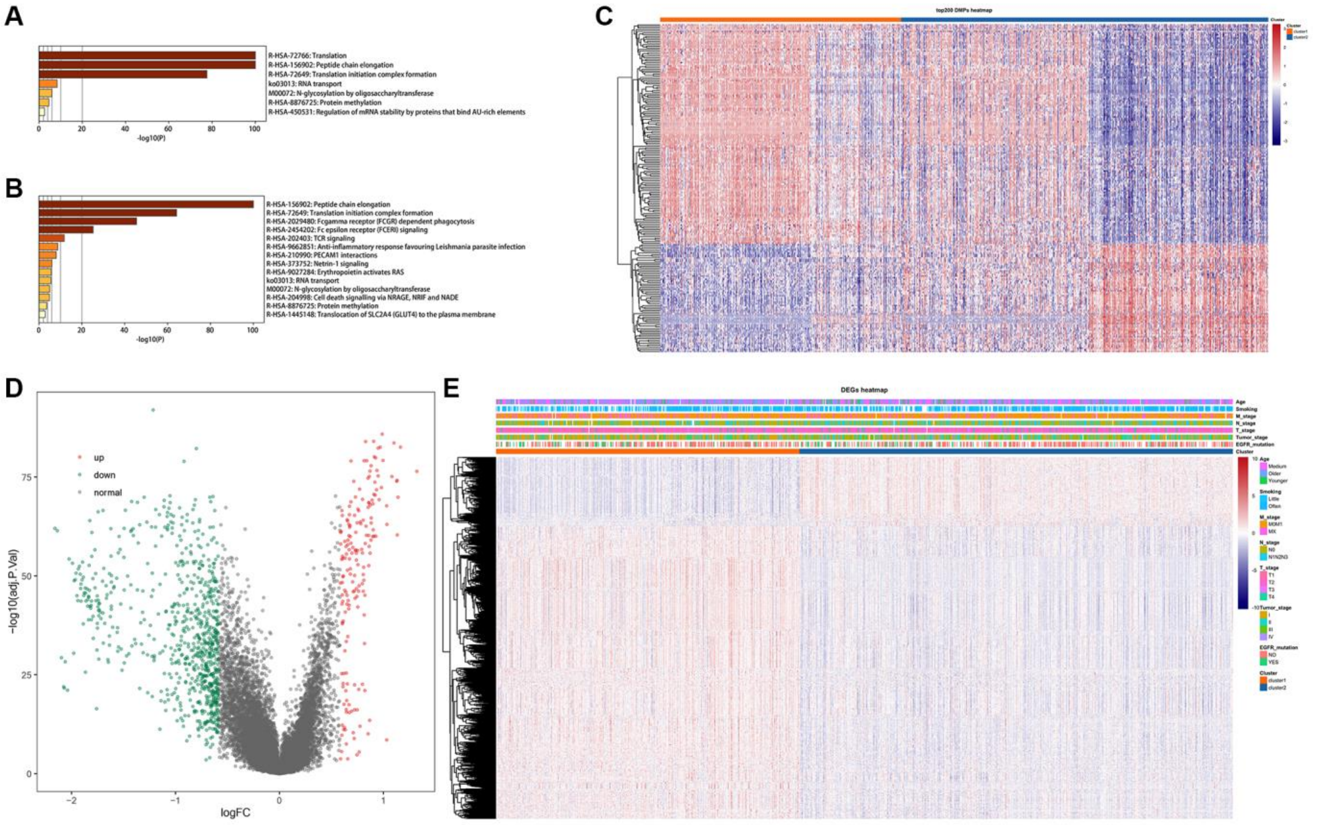

Supplementary Figure 5. (**A**) Pathway enrichment analysis results of the cluster 1. (**B**) Pathway enrichment analysis results of the cluster 2. (**C**) Identification results of differential methylation sites among characteristic subtypes: z-score heatmap of the top200 differential methylation sites. (**D**) Volcano map of differentially expressed genes. (**E**) Z-score heatmap of differentially expressed genes.
